# Decoding Green Consumption Behavior Among Chinese Consumers: Insights from Machine Learning Models on Emotional and Social Influences

**DOI:** 10.3390/bs15050616

**Published:** 2025-05-01

**Authors:** Ying Lu, Sang-Do Park

**Affiliations:** 1 Department of International Commerce and Business, Konkuk University, Seoul 05029, Republic of Korea; 2 Department of International Trade, Konkuk University, Seoul 05029, Republic of Korea

**Keywords:** green consumption behavior, machine learning models, emotional influence, eco-labels and green brands, social norms and sustainability

## Abstract

This study examined the diverse factors influencing green consumption behavior among Chinese consumers through a comprehensive, data-driven approach that integrated multiple machine learning models, including Gaussian naïve Bayes, K-nearest neighbor, multilayer perceptron, and XGBoost models. By analyzing emotional, product-related, cultural, social, and personal dimensions, this research identified key determinants that shape consumer engagement in sustainable consumption. Unlike conventional studies that rely on linear models or survey-based analyses, this study leveraged machine learning to uncover complex, nonlinear interactions between these factors. The findings reveal that emotional drivers, particularly guilt and pride, play a pivotal role in green consumption decisions, while cultural and product-related factors also exhibit significant influence. This study contributes methodologically by employing a multidimensional, multilevel analytical framework, enhancing the robustness of the findings. Furthermore, the results underscore the importance of policy and marketing strategies that effectively target emotional and social influences to cultivate a sustainable consumer culture. These insights provide actionable recommendations for policymakers and businesses seeking to promote green consumption and advance global sustainability efforts.

## 1. Introduction

With the continuous progress of human society, environmental issues, such as ecological degradation, resource scarcity, and frequent extreme weather events, have become increasingly prominent. People have gradually realized that while high-energy-consumption production methods have driven social progress, they also pose a serious threat to the environment (Huang et al., 2021; C. Wang et al., 2020; P. Wang et al., 2023). To address the challenges of climate change, governments worldwide have endeavored to integrate social development into environmental protection to achieve a sustainable economic model (Debbarma & Choi, 2022). In 2008 and 2016, the Organization for Economic Cooperation and Development (OECD) proposed that consumers should be considered the major driving force of sustainable development given that they account for more than 60% of final consumption in OECD countries (OECD, 2023). In other words, changes in consumer behavior need to be emphasized as a major driving force of global green growth. During the same period, China emerged as a rapidly growing consumer market in the global economy. Just as the massive production capacity and supply chains in China have driven global economic growth, China’s level of green consumption exerts a significant global influence that cannot be overlooked. Notably, the goals of achieving a carbon peak by 2030 and carbon neutrality by 2060 signify the shift in China from quantitative to qualitative growth at the macro level (X. Zhao et al., 2022), with a key strategic focus on promoting green consumption. Green consumption in China is being robustly advanced through top-down and bottom-up approaches, which serves as a healthy catalyst for reshaping global demand and supply chains. This initiative holds the potential to play a positive role in global environmental protection and addressing climate change. This underscores the critical importance of paying attention to green consumption in China.

In response to these policy changes, numerous researchers have conducted studies to identify the factors that influence the green consumption behavior of residents with the objective of gaining an in-depth understanding of these behaviors and promoting their adoption (Akhtar et al., 2021; Sreen et al., 2018; Y. Chen et al., 2021; Munerah et al., 2021; Leonidou et al., 2015; Chaudhary & Bisai, 2018). Based on previous studies, the choice of green consumption is evidently influenced by external environmental factors, income levels, and the internal emotions of consumers who have diverse personality traits (Chekima et al., 2016; Liobikienė et al., 2016; Paul et al., 2016; Y. Joshi & Rahman, 2015). However, the existing research mainly focuses on the impact of individual factors on green consumption behavior by primarily analyzing and discussing green consumption behavior using the theory of reasoned action (Ajzen, 1980) and the theory of planned behavior (TPB) (Ajzen, 1985). In reality, a combination of many factors typically influences green consumption behavior, and complex interactions occur among these factors, which may produce synergistic or offsetting effects under specific conditions. Detecting these effects is difficult through a study of individual factors alone (Sharma et al., 2023). Machine learning plays a crucial role in analyzing the complex interactions among multiple factors that influence behavior. This is because, compared to traditional models such as the TPB, machine learning models implement a more efficient variable-processing procedure. Specifically, the TPB assumes linear relationships between factors, which may fail to capture complex nonlinear interactions. In contrast, machine learning models can directly learn intricate relationships between inputs and outputs from data without requiring predefined linear assumptions. Machine learning models can handle a large number of variables, identify potential factors that significantly impact green consumption behavior, and more accurately model the nonlinear relationships among variables. Therefore, the current study used modeling and quantitative methods and employed machine learning to examine the green consumption behavior of Chinese consumers from a comprehensive perspective and to enhance the reliability of the research from an objective standpoint.

Importantly, we do not intend to position machine learning (ML) as a theoretical framework that is a substitute for established behavioral theories such as the TPB. Instead, we adopted ML as an advanced analytical method to complement the TPB framework. While the TPB provides a robust theoretical foundation for explaining green consumption behavior, it often assumes linear relationships among variables and may fail to capture complex, nonlinear interactions. ML techniques enable us to address these limitations by uncovering hidden patterns and enhancing the predictive accuracy of TPB-based constructs. This methodological approach aligns with recent research efforts that have integrated theory-driven variables with data-driven techniques to improve behavioral predictions (Azad et al., 2023). To achieve this goal, this study addressed the following key research questions:

RQ1: How do different consumer segments exhibit distinct patterns of green consumption behavior based on the frequency of, monetary spending on, and recency of purchases?

RQ2: Which factors exert the most significant influence on green consumption behavior, and how do their relative importance and interactions vary across different machine learning models?

By addressing these questions, this study extended prior research by integrating machine learning methodologies to uncover complex relationships among influencing factors and validate their predictive power across multiple analytical models. The integration of multiple machine learning models not only ensured the robustness of the analysis but also revealed intricate interdependencies among key influencing factors, addressing the limitations of traditional linear models. Furthermore, by demonstrating the effectiveness of artificial intelligence in analyzing nonlinear relationships and addressing class imbalance issues, this study advanced the methodological framework of consumer behavior research. These contributions provide both theoretical and practical implications for sustainability research, policy formulation, and green marketing strategies. The remainder of this paper is structured as follows. Section 2 provides a review of the four major categories of factors that influence consumption behavior. Section 3 introduces the modeling and data collection and cleaning processes. Section 4 presents the results of the analysis. Section 5 discusses the conclusions and implications of the research.

## 2. Literature Review

### 2.1. Cultural and Social Factors

In general, the decision-making process regarding green consumption is complex due to the influence of various factors (Yang et al., 2023). As external intervening factors, the social environment and culture significantly influence consumer behavior (S. Wang et al., 2016). To provide conceptual clarity in this study, we categorized the key socio-cultural variables measured as follows. We define “social norms” as the perceived expectations and pressures from others that influence an individual’s behavior (Ajzen, 1985). In contrast, “social identity” refers to an individual’s sense of belonging to a particular group, such as an environmentalist community, which can internally motivate green consumption behavior (Fielding et al., 2008). “Political ideology” pertains to an individual’s broader belief system regarding governmental and regulatory approaches to sustainability issues, reflecting cultural-level orientations toward environmental responsibility (Casper et al., 2021). Accordingly, social norms and social identity are treated as social factors because they arise from interpersonal relationships and group affiliations, whereas political ideology is classified as a cultural factor due to its connection to societal value systems and political culture. This classification provides a conceptual basis for understanding the diverse socio-cultural influences on green consumption behavior. X. C. Wei et al. (2017) reported that various factors, including economic benefits, product diffusion costs, and environmental losses, influence consumer decision-making interactions during the production diffusion process. However, Hong et al. (2021) found that regulatory subsidies can effectively encourage consumers to adopt green products. Meanwhile, Li and Gao (2022) discovered that early-stage penalties more substantially impact corporate green behavior than subsidies do. Therefore, the regulatory policies of the government play a crucial role in promoting green consumption behavior; however, monitoring and guiding consumers and businesses toward green behavior require further consideration. In information policy research, the management of social norms is considered a particularly effective method for encouraging environmentally friendly behaviors such as a reduction in household energy use and the recycling of waste (Allcott & Kessler, 2019; Nyborg et al., 2016). According to a number of researchers, when individuals perceive that the majority of people are consuming green products, they tend to opt for more environmentally friendly options (Demarque et al., 2015; H. Kim et al., 2012; Sparkman & Walton, 2017). Doherty and Webler (2016) argued that social norms also influence environmental behavior when specific individual actions (e.g., recycling) significantly impact emissions reduction. Moreover, social norms can effectively promote behavior change in consumer-relevant areas (Castro-Santa et al., 2023). Currently, the existing empirical evidence on the promotion of the shift toward green consumption among consumers through social norms primarily focuses on explicit norm communication (Castro-Santa et al., 2023). However, explicit norm communication is generally considered impractical, and countless products on the market are unable to comply with regulatory standards in the absence of corresponding policies for specific products. To investigate the relationship between green consumption and social norms, the majority of researchers employ variables with high degrees of dimensionality.

### 2.2. Green Product Factors

Achieving sustainable development is dependent on green product research and consumption (Kikuchi-Uehara et al., 2016; Liobikienė et al., 2016). Green products exhibit superior environmental performance throughout their manufacturing, use, and disposal processes compared with traditional products (Peattie, 1995). They can aid in the reduction of pressure on finite resources and slow down environmental degradation, which contributes to the harmonious development of society, benefits the economy, and helps protect the environment. Therefore, understanding the factors that influence consumers when selecting green products over other alternatives is crucial for the promotion of these products (Lv & Li, 2021; de Medeiros & Ribeiro, 2017). Researchers in the fields of marketing and engineering generally propose that the attributes of products play a critical role in driving green consumption (Diego-Mas et al., 2016; Gruber et al., 2014). By incorporating environmentally friendly characteristics and environmental information into the design, production, and marketing of green products, consumers can easily identify and select products that meet their environmental needs. This approach aids in increasing the market share of green products and creates added opportunities for the development of the environmentally friendly industry.

Regarding marketing, X. Zhang and Dong (2020) categorized product attributes into marketing and nonmarketing attributes. Marketing attributes included eco-labels, information credibility, promotions, and sales channels, while nonmarketing attributes included the product uniqueness, price, performance, country of origin, and health attributes. The results demonstrated that when purchasing green products, consumers were mainly concerned about nonmarketing attributes such as the availability of health attributes, product quality, packaging, and source, while marketing attributes exerted a relatively smaller impact. On the contrary, Xin and Long (2023) argued that eco-labels, as a powerful tool for promoting green consumption, provide consumers with environmental information about products. Meanwhile, the International Organization for Standardization (ISO, 1999) explicitly stated that environmental claims and labels should provide accurate and meaningful information to assist consumers in making environmentally responsible and sustainable purchasing decisions. Government and industry organizations in numerous countries and regions have implemented various environmental standards and certification mechanisms to promote green consumption and production and to ensure the transparency and veracity of product information provided to consumers. Therefore, comprehending the influence of eco-labels on the attitudes and behaviors of consumers regarding renewable energy, climate change, and green consumption is essential (Taufique et al., 2017).

### 2.3. Personal Factors

Personal characteristics frequently influence the green consumption behavior of consumers (Khaniwale, 2015). As people possess distinct personalities and psychological traits, the perspectives on green consumption vary, which results in diverse means of engaging in green consumption (Yakup & Jablonsk, 2012). Numerous researchers have endeavored to explain and identify the factors that influence green consumer behavior. For example, Bazzani et al. (2017) emphasized the openness, conscientiousness, extraversion, agreeableness, and neuroticism traits of consumers while investigating purchasing preferences. Suganya and Beena (2017) acknowledged this point of view and asserted the significant role of personal factors in the formation of consumer purchasing behavior. Meanwhile, Laroche et al. (2001) pointed out that individuals with green values tend to be more willing to engage in green consumption, because they are willing to pay extra for green products. However, the possession of green values does not necessarily indicate that consumers will engage in long-term green consumption. The reason for this is that the values of self-efficacy and self-transcendence also influence the green consumption behavior of consumers. Subsequently, Cannon and Rucker (2022) further explored the effects of self-efficacy and self-transcendence values on consumer behavior and proposed that self-efficacy can positively and negatively influence consumption behavior. Moreover, self-transcendence values can exert positive and negative impacts in certain situations, and the two influencing factors may yield similar results.

### 2.4. Emotional Factors

Emotions exert a substantial effect on green consumption behavior (Carrus et al., 2008). Differently to other emotions, guilt, pride, and awe emerge from personal or societal norms based on moral standards, which defines them as self-conscious emotions (Tracy & Robins, 2004). Guilt is “a general expectation of self-punishment for violating, anticipating a violation, or failing to meet internalized moral standards” (Kayal et al., 2018). According to Burnett and Lunsford (1994), the genesis of guilt emotions is contingent on two conditions. First, the behavior of people must contradict their inner moral guidelines; second, self-esteem must be diminished. In contrast, pride is a positive emotion that derives from a positive self-evaluation of one’s behavior (Lewis, 2008) and is largely dependent on self-awareness, self-evaluation, and self-reflection (e.g., J. E. Kim & Johnson, 2013; Haidt, 2003). According to Kristjánsson (2003), individuals experience pride when they or individuals with whom they identify achieve valuable accomplishments. Based on different attributions of pride, scholars distinguish between the positive and negative aspects of pride as two distinct dimensions. Lastly, people experience awe when they encounter something that significantly exceeds their current knowledge and existing mental structures (Keltner & Haidt, 2003). As a typical self-transcendent emotion, awe can extend an individual’s perception of time, foster a sense of relatively abundant time, and inspire a greater love of life, which results in high levels of life satisfaction (Rudd et al., 2012). Additionally, it can broaden the self-concept of an individual, which extends it to the external world (Van Cappellen & Saroglou, 2012).

In conclusion, various factors influence green consumption behavior. The current study classified these micro and fragmented factors into four major categories based on characteristics, namely, cultural and social, green product, personal, and emotional factors. Representative influencing factors were selected from each major category.

## 3. Data and Methodology

This section presents the overall framework of the research methodology. Data were divided into two major categories, namely, influencing factors (factors) and behaviors. First, the data of these influencing factors were normalized, and the entropy weight method was used to calculate the factor scores, which generated feature data. The data on green consumption behavior were then log-smoothed and normalized, and the first integer neighbor clustering hierarchy (FINCH) clustering method (Sarfraz et al., 2019) was used to obtain behavior categories, which served as label data. It should be clearly stated that the primary purpose of employing the FINCH clustering technique for the unsupervised clustering of consumer behavior was to objectively segment the data samples based on their distribution, thereby avoiding the potential subjective biases that could be introduced by manual classification. This clustering method itself was not intended to reveal causal relationships between variables. In our study, the causal relationships between variables were uncovered through the use of classification models, such as XGBoost and the KNN. These classification models learned from the labels generated by clustering to assess the impact of different factors on green consumption behavior and their interrelationships. Four machine learning models, namely, Gaussian naïve Bayes (Gaussian NB) (Smith & Johnson, 2018; L. Wang & Chen, 2020), K-nearest neighbor (KNN) (Lee & Park, 2019; H. Kim & Choi, 2021), multilayer perceptron (MLP) (R. Zhang & Li, 2017; Nguyen & Wang, 2020), and XGBoost (Brown & Davis, 2019; Patel & Shah, 2021) models, were trained, and the models underwent sensitivity analysis.

The primary advantage of this framework design was its incorporation of advanced data processing techniques, including the entropy weight method and FINCH clustering, which collectively enhanced the data quality, interpretability, and analytical rigor. By leveraging these methodologies, the framework ensured a more robust representation of the data variability and underlying structures, thereby improving the precision and reliability of the derived insights. Furthermore, the integration of these approaches strengthened the scientific validity of the results by mitigating potential biases, optimizing feature weighting, and facilitating more accurate classification and pattern recognition within the dataset.

Second, the advantages of different models were comprehensively considered using multiple machine learning models for training, which enhanced the credibility of the research conclusions. Moreover, by performing sensitivity analysis on the models, an in-depth evaluation of the influence of different factors on green consumption behavior was possible, which provided a basis for further data interpretation.

**Figure 1:**
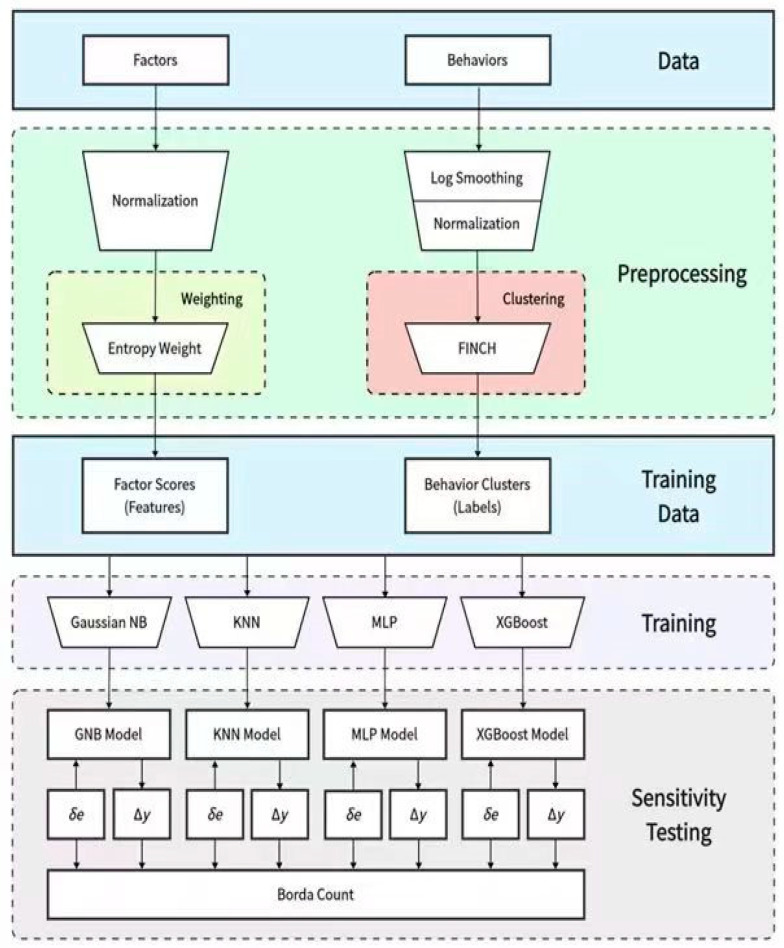
Research procedure.

The framework of this study was implemented based on Python version 3.11, fully leveraging its powerful data processing and machine learning libraries to conduct data preprocessing, model training, and result analysis. Specifically, the data preprocessing utilized efficient data analysis toolkits such as NumPy and Pandas. The FINCH clustering employed the Finch library, while the development of the classification models called upon advanced machine learning libraries like Scikit-Learn and XGBoost. Python’s high flexibility and rich open-source ecosystem make it an ideal choice for tackling complex data analysis tasks. (See Figure 1).

### 3.1. Survey Instrument Design

This study employed a structured questionnaire to collect data on the factors influencing green consumption behavior among Chinese consumers. The instrument was developed based on the previous literature and adapted to ensure content validity. The questionnaire consisted of four major parts. The first part was an introduction, which was primarily intended to inform respondents about the purpose of the survey. The second part was the main content of the questionnaire, which focused on the factors that influence green consumption behavior. The third part then collected the basic information of the respondents, including their gender, education, income, and occupation. The fourth part collected data on the amount of money spent on green consumption, the frequency of green consumption, and the timing of their earliest and most recent green consumption activities. In detail, the key variables used in this study were categorized into four major dimensions: first, cultural and social factors: the social norms, social identity, and political ideology that shape consumer behavior; second, green product-related factors: perceptions of eco-labels, green branding, and sustainable product attributes; third, personal factors: self-efficacy, ecological values, and nature connection, which influence individual attitudes toward sustainability; and finally, emotional factors: self-conscious emotions such as guilt, pride, and awe, which play a crucial role in green consumption decisions. Each variable was measured using a five-point Likert scale (1 = strongly disagree, 5 = strongly agree), and a reliability analysis was conducted to ensure the consistency of the measurement items (see Appendix A).

### 3.2. Data Collection and Source

A questionnaire was distributed to 500 Chinese consumers on the mainland to collect the necessary information. The survey was conducted through a professional survey agency, which employed random sampling techniques to distribute questionnaires widely among the entire population of Chinese consumers. The data for the study were collected between 16 March 2024 and 8 April 2024. Anomalous questionnaire data were considered invalid and excluded; for example, if there were unusual response times or if the records showed that the most recent behavior occurred before the first, this was deemed a data error and removed to ensure data and analysis quality. After excluding 130 invalid questionnaires, we obtained 370 valid responses. Table 1 and Table 2 illustrate this information.

### 3.3. Reliability and Validity

This study used exploratory factor analysis to test the reliability and validity of the questionnaire data. SPSS 29.0 was employed to analyze the reliability of the entire sample and each influencing factor to verify whether or not the responses were consistent across survey items. Table 3 demonstrates that the Cronbach’s alpha value for each factor exceeded the critical threshold of 0.7. These results indicate that the scale design was reasonable, and the questionnaire data exhibited high reliability. Using the Kaiser–Meyer–Olkin (KMO) test and Bartlett’s test of sphericity, the KMO value of the questionnaire data was found to be 0.948, with Bartlett’s test of sphericity reaching significance (*p* < 0.001). These results indicate that the questionnaire items corresponding to the latent variables had a high correlation and were suitable for factor analysis, which met validity standards.

### 3.4. Data Preprocessing

This study transformed the collected data to better fit the machine learning models. First, data smoothing and standardization were performed to smoothen the data distribution. For the data on the influencing factors, the study used the entropy weight method to weight each item within the influencing factors, which generates features for different items within each factor. For data on green consumption behavior, the study employed the FINCH clustering method (Sarfraz et al., 2019) to classify the green consumption behavior of consumers on the basis of data characteristics to avoid the influence of human intervention on the classification results. Finally, the categories obtained from clustering were used as labels for green consumption behavior, and they were combined with the factor scores to form the training data for machine learning.

#### 3.4.1. Entropy Weight Method

The entropy weight method determines the weight of each item within its corresponding factor by calculating the information entropy of each item, which reflects the degree of data dispersion. The smaller the entropy value of an indicator, the more concentrated the information and the greater the weight. The formula for calculating the weight is as follows:

$$w_{j}=\frac{1-H_{j}}{n-\sum_{j=1}^{n}H_{j},}$$

where $H_{j}=-k\sum_{i=1}^{m}p_{ij}\ln\left(p_{ij}\right)$ represents the information entropy of each item, and the proportion $p_{ij}$ represents the weight of each item within its corresponding factor. The entropy weight method is highly objective and entirely based on data, which reduces the impact of human factors.

#### 3.4.2. FINCH Clustering

FINCH clustering involves an efficient and parameter-free clustering algorithm (Sarfraz et al., 2019). It is based on the connection of neighboring points and recursively pairs each data point with its nearest neighbor, which identifies the connected components to construct a hierarchical clustering structure. Its major advantage lies in its ability to automatically determine a clustering structure without presetting parameters (e.g., the number of clusters) and with low algorithm complexity and a certain degree of interpretability.

Specifically, for a given dataset, $X=\{x_{1},x_{2},\ldots ,x_{n}\}$, the nearest neighbor $K_{i}^{1}$ of each data point, $x_{i}$, is calculated, and an adjacency matrix, A, is constructed, where

$$A(i,j)=\left\{\begin{array}{c} 1,\;if\;j=K_{i}^{1}\;or\;K_{j}^{1}=i\;or\;K_{i}^{1}=K_{j}^{1}\;\\ 0,\;\mathrm{otherwise} \end{array}\right.$$

Based on the adjacency matrix A, this study identified the connected components. Each connected component was considered a new data point, the nearest neighbor relationships were recalculated, and new connected components were identified. This process was repeated until no new merges occurred or the preset hierarchical depth was reached.

### 3.5. Machine Learning

Given the small scale of the dataset and the difficulty of accurately establishing the relationship between influencing factors and green consumption behavior, this study selected four machine learning methods, namely, the Gaussian NB, KNN, MLP, and XGBoost methods. The Gaussian NB model is a probabilistic model based on Bayes’ theorem and the assumption of feature independence (Kamel et al., 2019); the KNN is an instance-based learning method that does lack a clear model but performs classification or regression based on the labels of the nearest neighbors (Guo et al., 2003). Meanwhile, an MLP is an artificial neural network model composed of multiple layers of neurons that are capable of learning complex nonlinear relationships (Popescu et al., 2009), and XGBoost is an ensemble learning method that uses the gradient boosting of decision trees and combines multiple weak classifiers to build a strong classifier (T. Chen & Guestrin, 2016). The implementation principles of these four methods differ, which enabled them to complement one another’s limitations and reduce the output bias. To enhance the robustness of the research conclusions, the study comprehensively analyzed the results of the four models to eliminate differences in the impact of the influencing factors on green consumption behavior due to model differences. In this manner, the study obtained more reliable research conclusions.

### 3.6. Local Sensitivity

This study employed local sensitivity analysis to analyze the influence of different factors on green consumption behavior within the models. Local sensitivity analysis assesses the extent of change in the model output as a result of small input changes, which reveals the sensitivity of the model to input features. Subsequently, the results of the local sensitivity analysis of the four models were synthesized using the Borda count method, which is a collective voting method for ranking the influence of each factor based on the ranking scores of the models, which leads to reliable conclusions (Lansdowne & Woodward, 1996).

## 4. Results

### 4.1. Clustering Results for Green Consumption Behavior

**Figure 2:**
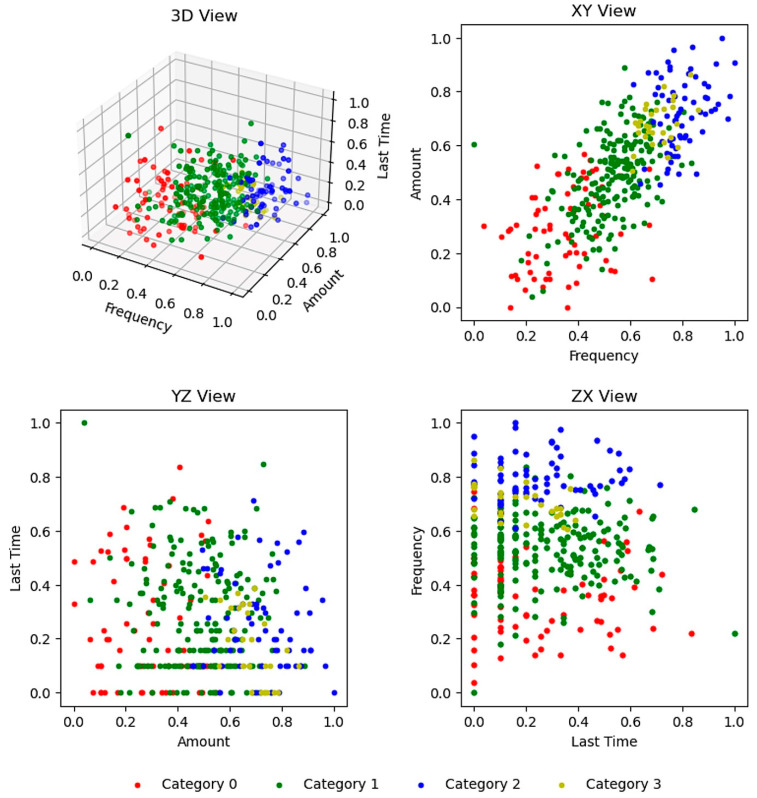
FINCH clustering results.

Without specifying the number of target categories, the FINCH algorithm clustered all samples into four categories after three iterations. For an intuitive visualization, this study log-smoothed and normalized the five-dimensional frequency, three-dimensional monetary, and one-dimensional recency data on green consumption behavior. The averages were then calculated and integrated into three dimensions, namely, frequency, monetary, and recency dimensions, based on the RFM (recency, frequency, and monetary) model. The RFM model is suitable for identifying customer purchasing behavior (J.-T. Wei et al., 2010; Ho et al., 2023) and could be used to segment green consumption behavior in our data. Each sample point was marked based on its category in this three-dimensional space, as shown in the 3D view in Figure 2.

Based on the 3D view in Figure 2, this study observed that the four categories clustered using the FINCH algorithm were clearly separated in space.

First, in the frequency and monetary dimensions, categories 0, 1, 2, and 3 exhibited a clear progressive relationship. The XY view indicates that the characteristics of the green consumption behavior of these categories can be summarized as “low frequency, low monetary”, “relatively low frequency, relatively low monetary”, “high frequency, high monetary”, and “relatively high frequency, relatively high monetary”. This classification differs from the traditional two-dimensional quadrant method, which classified consumption behavior into “low frequency, low monetary”, “low frequency, high monetary”, “high frequency, low monetary”, and “high frequency, high monetary”. Specifically, this study found that nearly no cases of “low frequency, high monetary” and “high frequency, low monetary” existed in actual green consumption behavior. Nevertheless, there was a clear linear relationship between the frequency of green consumption behavior and the monetary value, indicating that a higher consumption frequency corresponds to a higher monetary value, and vice versa.

Second, the time since the last green consumption behavior did not point to a significant linear relationship between the frequency and monetary dimensions, which is evident in the YZ and ZX views. The distribution of items in both views displays a clear boundary with changes in the *monetary* and frequency dimensions, which indicates a significant impact of these two dimensions. However, the distribution was relatively uniform with changes in the time since the most *recent* green consumption behavior. The only exception was that category 3 exhibited a recent occurrence of green consumption behavior with nearly no distribution in the “Last Time” interval greater than 0.4.

In summary, the overall characteristics of the four categories clustered using the FINCH algorithm can be summarized as follows: “low frequency, low monetary”, “relatively low frequency, relatively low monetary”, “high frequency, high monetary”, and “relatively high frequency, high monetary, recent green consumption behavior”.

### 4.2. Machine Learning Results

This study trained and tested the four abovementioned machine learning models on the data. Table 4 presents their performance in terms of their accuracy and F1 score.

The application of four different machine learning models in this study provided insights into the patterns of green consumption behavior. The comparative analysis of these models suggested that using multiple approaches can help identify consistent patterns in the data.

Among the four models, XGBoost achieved the highest accuracy (0.80) and the best F1 score (0.73). These results suggest that XGBoost may be more effective in predicting factor categories and balancing precision and recall, particularly in addressing class imbalance issues. However, given the limited sample size, these differences in performance should be interpreted with caution. Further validation with larger datasets is needed to confirm these findings. The KNN also performed well, with an accuracy of 0.78 and an F1 score of 0.71. The Gaussian NB model demonstrated a comparable F1 score (0.71) but had slightly lower accuracy (0.77), while the MLP had the lowest F1 score (0.63) among the models.

By integrating multiple machine learning models, this study not only verified the robustness of its findings but also enhanced the depth of analysis regarding variable interactions. Different models capture different aspects of data complexity, and their comparative performance helps identify patterns that might not be evident when relying on a single approach. This methodological diversity ensures that the relationships among key factors are examined from multiple perspectives, contributing to a more nuanced and comprehensive understanding of the underlying dynamics.

Moreover, using multiple models allows for the better validation of analytical results. If the findings remain consistent across different models, confidence in their reliability increases. Conversely, discrepancies in model performance highlight potential complexities or nuances in data relationships, prompting further investigation. Therefore, the use of the XGBoost, KNN, Gaussian NB, and MLP models not only strengthened this study’s analytical framework but also provided valuable insights into the interplay among key variables, ultimately improving the interpretability and generalizability of the research findings.

### 4.3. Sensitivity Analysis Results

**Figure 3:**
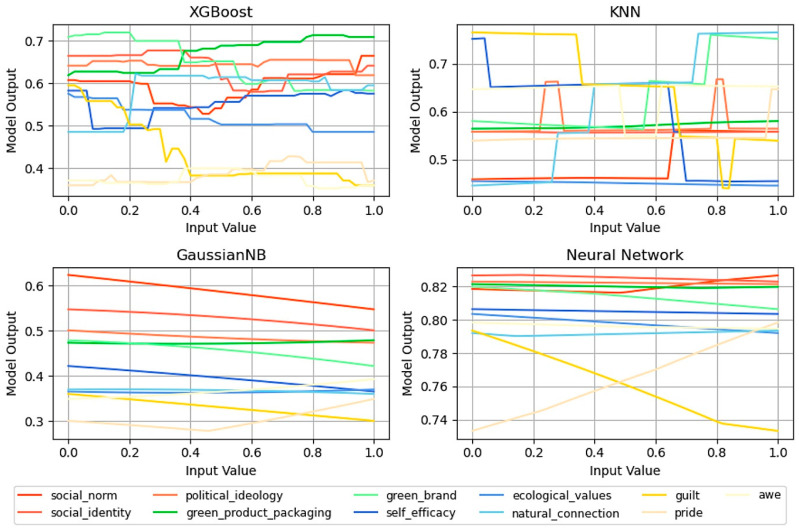
Sensitivity curves for XGBoost, KNN, Gaussian NB, and MLP models.

**Figure 4:**
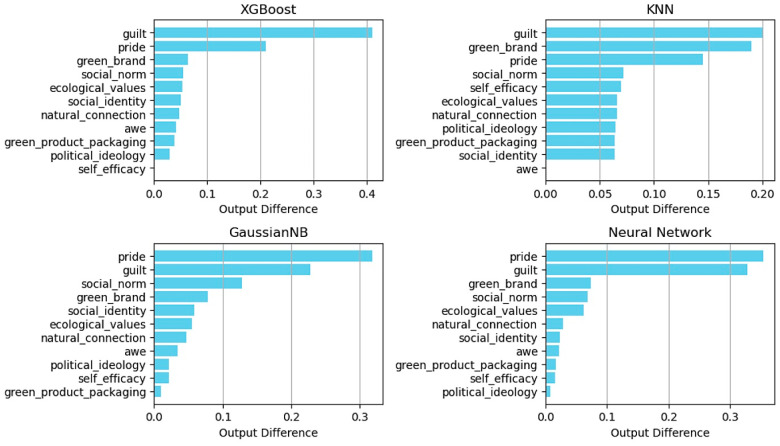
Sensitivity ranking of XGBoost, KNN, Gaussian NB, and MLP models.

Figure 3 presents the sensitivity analysis curves of the different factors for various models. By observing the fluctuations in these curves with changes in the input parameters, this study visually reflected the influence of these factors on the model output. Specifically, curves with more significant fluctuations indicated that the factor exerted a stronger influence on green consumption behavior. To further quantify and rank this influence, we calculated the absolute value of the change in each curve. Figure 4 illustrates the results of this comprehensive analysis, which highlighted the relative importance of each factor and provided intuitive evidence for understanding the key elements that drive green consumption. Sensitivity analysis not only quantifies the specific impacts of various factors on model outputs but also uncovers the intricate nonlinear relationships among factors under multifactor interactive conditions, which can be visually observed in Figure 3. This complex interplay of multiple factors is often not captured in single-factor studies, as they typically involve the strict control of other variables, thereby overlooking the mutual influences between variables.

The results of the research data analysis imply that the emotional factors guilt and pride were notably prominent among all factors, and they became the most critical factors that influenced green consumption behavior. Specifically, the XGBoost and KNN models identified guilt as the primary influencing factor, while in the Gaussian NB and MLP models, it ranked as the second most important. Conversely, pride occupied the primary position in the Gaussian NB and MLP models, while it ranked second or third in the XGBoost and KNN models. This finding indicates that regardless of the prediction model, guilt and pride remained the two core elements that drove the predictive performance of the model.

The influence of a green brand was widespread, as it ranked among the top three in the XGBoost, KNN, and MLP models and only fell to fourth place in the Gaussian NB model, which demonstrates its consistent significant role across models. Additionally, social norms and ecological values were highly rated in all four models, in which social norms consistently ranked in the top four and ecological values were mostly in fifth or sixth place, which indicates a broad recognition of their importance in guiding consumer behavior.

The roles of social identity and natural connection in the models were intermediate, with social identity generally ranking between fifth and seventh, except in the KNN model, where it ranked slightly lower. Natural connection was consistently positioned in sixth or seventh place. Alternatively, factors such as awe, green product packaging, political ideology, and self-efficacy ranked relatively low in all models, which implies that these factors exerted a relatively weaker direct influence on green consumption behavior.

In summary, the four models exhibited notable consistency in addressing these factors with slight differences in individual influencing factors, which were primarily dependent on the unique characteristics of the model structure and algorithm mechanism.

**Figure 5:**
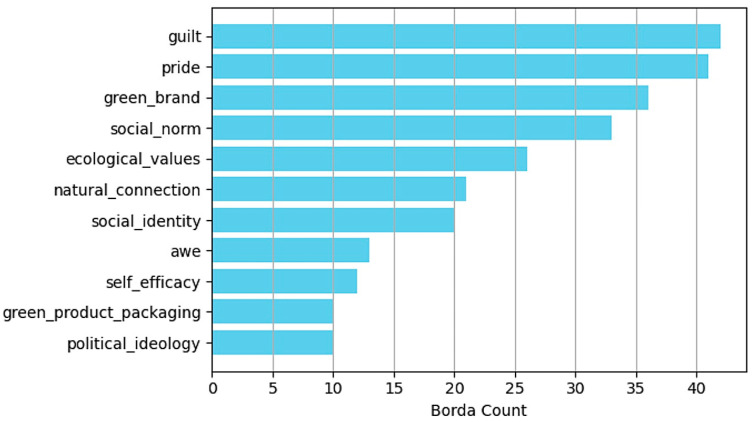
Comprehensive evaluation results for XGBoost, KNN, Gaussian NB, and MLP models.

Figure 5 depicts the influence of different factors on the overall green consumption behavior by ranking the comprehensive evaluation results of the four models using the Borda count method. This comprehensive analysis indicates that emotional factors (guilt and pride) exerted the most significant influence on green consumption decisions, which highlights the powerful role of emotions in driving behavior. The green product factor of green brands was also crucial, because brand identity can significantly influence consumer choices. Cultural and social factors, particularly social norms, exerted a considerable impact on individual behavior, while political ideology exerted a relatively minor direct impact. In the realm of personal factors, ecological value and natural connection exerted a significant influence on green consumption behavior, while self-efficacy played a relatively weak role.

## 5. Discussion

### 5.1. Academic Implications

This study makes meaningful academic contributions by advancing both the theoretical and methodological understanding of green consumption behavior. Theoretically, our findings enrich the existing literature by identifying and validating key drivers of environmentally responsible consumption. Specifically, the results underscore the pivotal role of emotional factors—particularly guilt and pride—in motivating green consumption. These self-conscious emotions, which reflect an individual’s internal moral framework, were consistently identified as significant predictors across all ML models employed in this study. This outcome reinforces prior research that highlights the influence of emotions in shaping consumer behavior and provides robust empirical evidence for the argument that moral emotions can drive sustainable consumption choices.

Beyond emotional influences, our analysis also emphasized the importance of green product-related factors, such as eco-labels and brand identity, in shaping consumer decision-making. The quantitative assessment of these attributes revealed that product characteristics not only affect consumers’ purchase intentions but also strengthen brand trust and loyalty in the context of green consumption. This finding aligns with established marketing theories that underscore the significance of brand image and perceived quality in influencing consumer behavior and extends their applicability to sustainable consumption practices.

From a methodological perspective, this study demonstrates the value of integrating ML techniques into the analysis of consumer behavior. Unlike traditional statistical models, which often assume linear relationships and have limitations in handling complex interactions, ML approaches can effectively capture nonlinear and multidimensional relationships among influencing factors. By employing four distinct ML models—the Gaussian NB, KNN, MLP, and XGBoost models—our research addressed the limitations of conventional analytical methods and enhanced the predictive accuracy and robustness of behavioral analysis. Importantly, we do not suggest that ML serves as an alternative theoretical framework to well-established behavioral models such as the TPB. Rather, we employed ML as an advanced analytical tool that complements and extends the TPB by uncovering hidden patterns and intricate interactions among variables that may otherwise remain undetected (Azad et al., 2023).

In addition, this study contributes to theoretical clarity regarding the role of socio-cultural factors in driving green consumption behavior. We explicitly differentiated and categorized social norms, social identity, and political ideology to avoid conceptual ambiguity. Social norms and social identity were defined as social factors because they originate from interpersonal relationships and group affiliations, while political ideology was treated as a cultural factor, reflecting broader societal values and belief systems. Our empirical analysis confirmed that social norms exert significant external pressure on consumers to conform to environmentally friendly behaviors, whereas social identity functions as an internal motivator linked to individuals’ sense of belonging to pro-environmental communities. The role of political ideology, although exhibiting a weaker direct effect in our findings, still provides meaningful insight into how broader cultural orientations may shape sustainability-related behavior, particularly in contexts characterized by strong policy interventions and homogenous political environments, such as China.

Taken together, these findings provide a comprehensive and nuanced understanding of the multidimensional factors influencing green consumption behavior. By integrating advanced analytical techniques with well-established theoretical constructs, this study contributes to the development of a more sophisticated methodological framework and offers valuable theoretical insights into the psychological, social, and cultural determinants of sustainable consumption. We believe that these contributions will serve as a useful foundation for future research and policymaking aimed at promoting environmentally responsible consumer behavior.

### 5.2. Policy Implications

From a policy perspective, this study provides valuable insights into how policymakers can effectively promote green consumption behavior among consumers. The findings emphasize the importance of addressing emotional factors, such as guilt and pride, which suggests that public campaigns and policies intended to evoke these emotions could be particularly effective. For instance, the promotion of messages that highlight the positive impact of green consumption on the environment or the negative consequences of a lack of engagement with such behaviors can leverage guilt and pride to drive consumer action. Policymakers can design interventions that tap into these emotions, such as increasing the public awareness of the benefits of green consumption or reminding consumers of the environmental damage caused by traditional consumption choices. In this manner, a more sustainable consumer culture can be fostered.

This study also underscores the critical role of green product-related factors, such as eco-labels and brand identity, in influencing consumer behavior. This aspect suggests that policies intended to enhance the visibility and credibility of eco-labels could play a crucial role in the promotion of green consumption. Governments can collaborate with industry stakeholders to standardize and regulate eco-labeling, which will ensure that these labels are trustworthy and easily recognizable by consumers. Furthermore, supporting the development and promotion of green brands through subsidies, certifications, and marketing support can strengthen consumer trust and increase engagement with sustainable products.

Furthermore, this study highlights the role of cultural and social factors, such as social norms and ecological values, as significant drivers of green consumption. Policymakers can leverage these insights by fostering a societal culture that values environmental responsibility. This goal can be achieved by conducting education and awareness campaigns that promote green behavior as a societal norm. By showcasing green consumption as a socially desirable and expected behavior, policymakers can harness the power of social conformity to encourage the widespread adoption of sustainable practices. Additionally, the integration of ecological education into school curricula and public information initiatives can help instill ecological values from a young age, which can lead to long-term changes in consumer behavior.

The findings also point to the importance of strengthening market intermediary organizations and legal and institutional environments to support green consumption. Thus, policymakers should focus on the creation of a supportive infrastructure for green businesses, such as by providing incentives for companies that adopt sustainable practices or penalizing those that do not. Strengthening the legal framework to protect green consumers, such as enforcing stringent penalties for false advertising related to eco-products, can further enhance consumer confidence and the willingness to engage in green consumption.

Lastly, this study suggests a two-way interaction between enterprise innovation and the external environment. In other words, policies should not only aim to influence consumer behavior but also support the innovation efforts of businesses. By providing support for the research and development of sustainable technologies and encouraging businesses to innovate greener products and services, policymakers can create a virtuous cycle in which consumers’ demand for green products drives further innovation, which, in turn, supports broader environmental goals.

### 5.3. Practical Implications

The practical implications are highly relevant for businesses, marketers, and other stakeholders that aim to effectively promote green consumption behavior. By understanding the various factors that drive consumers toward sustainable choices, companies can refine their strategies to better align with consumer motivations and foster an environmentally conscious marketplace.

First, one of the key findings was the significant impact of emotional factors, particularly guilt and pride, on green consumption behavior. For businesses, this finding presents a valuable opportunity to design marketing campaigns that tap into these emotions. Thus, companies can create advertisements that emphasize the environmental benefits of their products, which makes consumers feel pride in their purchase decisions. Alternatively, campaigns that highlight the negative environmental impact of non-green alternatives can invoke a sense of guilt, which could motivate consumers to make more sustainable choices. For instance, a company could showcase how much waste or carbon emissions are saved by selecting their product over a conventional option. This approach can be particularly effective in industries, such as the food and beverage, fashion, and home goods industries, in which consumers are increasingly aware of their environmental footprint. Moreover, brands can foster a sense of community centered on green consumption by creating loyalty programs or customer engagement initiatives that reward sustainable behaviors. For example, they could offer discounts, special promotions, or public recognition to customers who consistently opt for eco-friendly products, which could establish a positive feedback loop in which consumers feel emotionally rewarded for their choices. These strategies not only drive sales but also strengthen brand loyalty by aligning with the values and emotions of consumers.

Second, this study highlights the importance of green product-related factors, such as eco-labels and brand identity, in influencing consumer behavior. Businesses should invest in the acquisition of credible eco-certifications and prominently display these labels on their products. Doing so not only provides consumers with the information they need to make environmentally responsible choices but also builds trust in the brand. Companies must ensure that their claims of sustainability are transparent, verifiable, and consistent, because misleading claims can lead to consumer distrust and damage to a brand’s reputation. To enhance brand trust, companies can also adopt a holistic approach to sustainability through the integration of environmentally friendly practices into the entire value chain, from sourcing raw materials to manufacturing processes and distribution. Effectively communicating these efforts through storytelling, transparency reports, and sustainability updates can further differentiate a brand in a crowded market. Moreover, businesses should consider investments in traceability and blockchain technologies to provide verifiable proof of green practices, which will appeal to increasingly discerning and informed consumers.

Third, social norms and ecological values are significant drivers of green consumption, as indicated by this study. Practically, businesses can capitalize on these insights by positioning green products as not only beneficial but also as the socially expected choice. Therefore, formulating marketing messages that align green consumption with social approval can create a powerful motivator. For example, messaging that emphasizes being part of a broad movement or community of environmentally conscious consumers can leverage the power of social conformity. This initiative can be achieved through influencer marketing, social media campaigns, or partnerships with eco-friendly organizations that resonate with target audiences. Furthermore, companies could consider engaging in corporate social responsibility (CSR) initiatives that align with ecological values. By participating in environmental campaigns, sponsoring green events, or contributing to sustainability causes, businesses can reinforce their commitment to the environment and enhance their appeal to consumers who prioritize ecological values. As such, CSR activities should be communicated effectively to the public to build brand affinity and loyalty among eco-conscious consumers.

Fourth, the findings underscore the importance of market intermediary organizations and the legal environment in promoting green consumption. Businesses, particularly small- and medium-sized enterprises, can benefit from participating in or forming alliances with market intermediaries such as green industry associations, sustainability hubs, and eco-friendly certification bodies. These intermediaries can provide valuable resources, including market insights, networking opportunities, and access to information on sustainability best practices. Additionally, companies should advocate for and engage in dialogues with policymakers to establish a supportive legal and regulatory environment for green products. This aspect includes lobbying for incentives, such as tax breaks, subsidies, or grants, for businesses that adopt sustainable practices. Moreover, companies could participate in public consultations and industry panels to help establish standards and regulations that align with industry capabilities and sustainability goals. By taking an active role in the development of a supportive market and regulatory infrastructure, businesses can not only enhance operational efficiencies but also contribute to broad and industry-wide changes that promote green consumption.

Fifth, another practical implication is the potential for businesses to use data analytics and consumer feedback to continuously innovate and refine green offerings. By leveraging machine learning and other data-driven approaches, companies can gain insights into consumer preferences, identify trends in green consumption, and develop products that meet the evolving needs of environmentally conscious consumers. For instance, businesses can analyze purchasing data to determine the eco-friendly features that are most valued by consumers and use this information to guide product development. Furthermore, incorporating customer feedback mechanisms, such as surveys, focus groups, or social media interactions, can provide valuable insights into the perceptions of consumers about the green initiatives and products of a company. This feedback can inform marketing strategies, product enhancements, and even the development of new green products that align more closely with consumer desires.

Finally, this study recommends that although short-term efforts to enhance internal innovation capabilities are essential, businesses must prepare for long-term engagement with green consumption trends. This notion involves not only the adaption of products and services to meet the current consumer demands but also the anticipation of future shifts in consumer behavior, the regulatory landscape, and technological advancements. Companies should invest in strategies for long-term sustainability that encompass product innovation and operational sustainability to remain competitive as the market for green products continues to grow. By positioning themselves as leaders in sustainability, businesses can build a resilient brand that appeals to a broad range of stakeholders, including consumers, investors, and employees, who value environmental stewardship. Long-term engagement also indicates adaptability and a readiness to pivot strategies in response to changes in the market or regulatory environment, which could ensure sustained success in the promotion of green consumption.

## 6. Conclusions and Limitations

This study systematically analyzed multiple factors that influence green consumption behavior through the comprehensive use of four machine learning models, namely, the and Gaussian NB, KNN, MLP, and XGBoost models. The results particularly underscore the significant roles of emotional dimensions (guilt and pride), green product dimensions (green brand), cultural and social dimensions (social norms), and individual dimensions (ecological values). The findings strongly indicate that various factors influence green consumption behavior, such that focusing solely on a single area is insufficient. Emotional responses play a major role in green consumption choices, while external green brand recognition significantly influences the purchasing decisions of consumers based on various factors such as brand trust and loyalty. Social norms drive individuals to adopt environmentally friendly behaviors, both from a broad perspective and regarding specific points, while ecological values support green consumption behavior through intrinsic environmental awareness and moral responsibility.

The innovation of this study lay in its use of a multidimensional and multilevel perspective; as such, it overcame the limitations of single-factor analysis and showcased the complex relationships that underlie green consumption behavior. Through cross-validation with multiple models, the study not only confirmed the general consistency of the influence of different factors but also highlighted the superiority of data-driven methods in behavior prediction, which provides a new perspective for research on green consumption behavior. This integrated analytical approach enriches the current understanding of green consumption behavior and offers policymakers and businesses additional comprehensive references to design more effective strategies and marketing plans for environmental promotion, which, thus, better advances the adoption and development of green consumption. By revealing these driving factors and their interactions, this study provides a solid theoretical foundation and practical guidance for future environmental practices.

Despite its achievements, this study has its limitations. First, the sample was concentrated on Chinese consumers, such that the global applicability of the conclusions requires cross-cultural validation. Second, although the study covered the majority of factors identified by previous research, it may have overlooked other factors. Finally, these findings should be interpreted with caution, as the overall performance differences were modest and may not have been statistically significant due to the limited sample size. The generalizability of these findings may be limited, and further research is needed to explore the potential influence of other factors on green consumption behavior. Thus, future research could expand the scope of the study to compare whether or not green consumption behavior varies among consumers across countries or regions under the influence of these factors. Further analysis could also investigate the effect of the interactions of these factors on green consumption behavior. Lastly, although the study discussed the impact of various factors on green consumption behavior in relation to different dimensions, it found that cases occurred in which the significance of multiple factors was comparable. This implies that despite our study focusing not on simple causal verification based on hypothesis testing but rather on predicting causality among variables and deriving interrelationships among the most influential ones, issues of reciprocal causality within the sample may still exist. In future research, a more in-depth investigation and additional exploratory analysis can be conducted by expanding the question design to employ a formative measurement model instead of a reflective measurement model during the variable-setting process and applying PLS-SEM.

## Figures and Tables

**Table 1:** Basic information of respondents.

				N (%) = 370 (100.0%)	
Variable	Category	N (%)	Variable	Category	N (%)
Gender	Male	45.41	Occupation	Civil Servant/Public Institution	10.54
	Female	54.32		Freelancer (e.g., Writer/Artist/Photographer/Tour Guide)	5.68
Age	Younger than 20	14.86		Service Industry	4.32
	20–30	35.14		Company Employee	35.95
	30–40	24.86		Businessman/Employer/Self-Employed	5.95
	40–50	12.97		Worker (e.g., Factory Worker/Construction Worker/Sanitation Worker)	5.68
	Older than 50	11.35		Professional (e.g., Lawyer/Engineer)	6.76
Income	Below RMB 3000/month	31.89		Student	23.78
	RMB 3001–6000/month	22.97	Education	High School or Below	23.51
	RMB 6001–9000/month	24.05		Associate Degree	24.59
	RMB 9001–12,000/month	12.43		Bachelor’s Degree	44.32
	More than RMB 12,000/month	8.11		Graduate (Master’s/PhD)	6.76

**Table 2:** Factors influencing green consumption behavior.

N (%) = 370 (100.0%)							
Frequency of Green Consumption (Times)		<5	<10	<20	<50	<100	≥100
	Purchase of Eco-Friendly Products	6.76	15.68	30	30	13.24	4.32
	Second-Hand Goods Transactions	44.86	28.65	17.57	8.11	0.81	0
	Low-Carbon Travel	5.68	10.54	22.16	42.16	15.95	3.51
	Waste Sorting	11.62	10	14.32	17.3	10.54	36.22
	Participation in Environmental Activities	57.84	22.97	13.51	5.68	0	0
Amount Spent on Green Consumption (RMB)		<500	<1000	<2000	<5000	<10,000	≥10,000
	Purchase of Eco-Friendly Products	20.81	15.14	20.27	23.78	11.35	8.65
	Second-Hand Goods Transactions	44.32	19.19	15.14	13.78	6.22	1.35
		<10	<50	<100	<200	<500	≥500
	Low-Carbon Travel	5.41	30.54	20.27	21.08	20.27	2.43
Lifecycle (Days)		<5	<10	<20	<50	<100	≥100
	Time Since First Green Consumption Activity	52.97	11.62	13.24	13.24	4.86	4.05

Lifecycle refers to the time span between the earliest and most recent green consumption activities.

**Table 3:** Feasibility and reliability verification of measurement tools.

Construct	Item	Factor Loading	Extraction	% of Variance	%	Cronbach’s α
Cultural and Social Factors	People around me, such as family, relatives, and friends, often engage in green behaviors.	0.75	0.715	5.948	10.814	0.966
	Most people around me believe that we should protect the environment through our own actions.	0.7	0.68			
	Behaviors that pollute the environment are condemned by most people around me.	0.784	0.724			
	The group I belong to can reflect that I am an environmentally conscious person.	0.703	0.688	4.535	19.06	0.788
	I consider myself part of an environmentalist group.	0.724	0.714			
	I am valuable within the environmentalist group.	0.734	0.714			
	Despite my small influence, I also contribute to environmental protection.	0.721	0.67			
	The government should enact relevant policies or laws to regulate people’s green consumption behavior.	0.763	0.717	4.139	26.584	0.845
	If relevant policies and regulations require green consumption behavior, I will actively cooperate.	0.781	0.739			
	To avoid fines from certain departments, I will reduce environmentally harmful behaviors.	0.737	0.682			
	Policies and regulations can provide guidance for the implementation of green consumption behaviors.	0.706	0.618			
Green Product-Related Factors	Green product packaging contributes to environmental protection.	0.797	0.746	3.805	33.502	0.848
	Green product packaging is an excellent example of eco-friendly packaging.	0.763	0.743			
	Green product packaging aligns with the environmental positioning of the product.	0.696	0.646			
	Green product packaging should feature green product labels.	0.797	0.743			
	The environmental benefits of green brands, such as energy-saving and zero emissions, attract me.	0.677	0.683	3.665	40.166	0.861
	The green manufacturing processes and green technologies of green brands are very appealing to me.	0.707	0.668			
	Green brands’ products do not harm the environment after disposal, which is very appealing to me.	0.692	0.635			
	Purchasing products from green brands is beneficial for the environment and society, which is very appealing to me.	0.731	0.729			
	The values of green brands promoting environmental benefits align with my beliefs.	0.613	0.606			
	I would feel regret if a brand does not have eco-friendly features.	0.685	0.649			
	The green corporate culture of the brand’s parent company inclines me towards it.	0.702	0.674			
	The brands of products I purchase have consistently been green brands.	0.684	0.651			
	I often recommend green product brands to relatives and friends.	0.646	0.66			
Personal Factors	I have confidence in implementing green consumption behaviors.	0.69	0.739	3.027	45.67	0.929
	I believe I have the ability to implement green consumption behaviors.	0.742	0.74			
	I believe that, as long as I am willing to make an effort, I can improve or address certain environmental issues.	0.68	0.728			
	If I encounter problems in implementing green consumption behaviors, I have confidence in finding solutions.	0.729	0.745			
	Natural resources are the foundation for human survival and development, and humans should reduce their consumption.	0.767	0.667	2.952	51.036	0.875
	Nature has inherent intrinsic value, and humans should adapt to and respect nature.	0.778	0.687			
	Human society must coexist harmoniously with nature to survive.	0.721	0.619			
	Economic development must proceed in parallel with environmental protection.	0.783	0.697			
	Harmony between humans and nature is a sign of social progress.	0.793	0.763			
	I believe that caring for nature and protecting the environment is important.	0.631	0.549			
	I feel connected to the surrounding natural world.	0.742	0.689	2.942	56.385	0.887
	I can perceive and appreciate the wisdom of other life forms.	0.772	0.773			
	I often feel an affinity with animals and plants.	0.761	0.786			
	I have a deep understanding of how my actions affect the natural world.	0.744	0.725			
	I feel that all residents of the Earth (both human and non-human) share a common vitality.	0.792	0.801			
Emotional Factors	If my failure to engage in green consumption leads to adverse environmental impacts, I feel guilty.	0.757	0.757	2.81	61.494	0.913
	If my failure to engage in green consumption leads to adverse environmental impacts, I feel remorse.	0.773	0.759			
	If my failure to engage in green consumption leads to adverse environmental impacts, I feel remorse.	0.743	0.781			
	If my failure to engage in green consumption leads to adverse environmental impacts, I feel ashamed.	0.735	0.747			
	I consider myself an environmentally conscious consumer.	0.723	0.715	2.795	66.576	0.895
	The green products I use effectively demonstrate that I am a responsible consumer.	0.662	0.727			
	I take pride in being an environmentalist.	0.621	0.674			
	I desire for my family and friends to see me as someone who cares about environmental issues.	0.688	0.691			
	If I purchase green products, I am very satisfied with myself.	0.646	0.666			
	I buy and use green products because they highlight my pro-environment personality.	0.618	0.661			
	Engaging in environmental activities is an important part of my life.	0.719	0.726			
	I feel awe towards nature.	0.714	0.675	2.152	70.488	0.922
	My surroundings are full of beauty.	0.703	0.692			
	I often seek patterns in the things around me.	0.788	0.774			
	I have many opportunities to appreciate the beauty of nature.	0.725	0.743			
	I seek certain experiences to challenge my understanding of the natural world.	0.793	0.780			
Kaiser–Meyer–Olkin Measure of Sampling Adequacy: 0.948 Bartlett’s Test of Sphericity: Approx. Chi-Square: 13,764.646; df: 1485; Sig. < 0.001						

**Table 4:** Gaussian NB, KNN, MLP, and XGBoost model evaluations for the classification of green consumer behavior.

	XGBoost	KNN	Gaussian NB	MLP
Accuracy	0.80	0.78	0.77	0.77
F1 Score	0.73	0.71	0.71	0.63

## Data Availability

The raw data supporting the conclusions of this article will be made available by the authors on request.

## Appendix A. Survey Instrument Design

**Variables**	**Dimensions**	**Measurement Items**	**Sources**
Cultural and Social Factors	Social Norms	People around me, such as family, relatives, and friends, often engage in green behaviors.	Gleim et al. (2013); Mancha and Yoder (2015); L. M. Zhao et al. (2015)
		Most people around me believe that we should protect the environment through our own actions.	
		Behaviors that pollute the environment are condemned by most people around me.	
	Social Identity	The group I belong to can reflect that I am an environmentally conscious person.	Cameron (2004); Dholakia et al. (2004)
		I consider myself part of an environmentalist group.	
		I am valuable within the environmentalist group.	
		Despite my small influence, I also contribute to environmental protection.	
	Political Ideology	The government should enact relevant policies or laws to regulate people’s green consumption behavior.	T. B. Chen and Chai (2010); Sujata et al. (2019)
		If relevant policies and regulations require green consumption behavior, I will actively cooperate.	
		To avoid fines from certain departments, I will reduce environmentally harmful behaviors.	
		Policies and regulations can provide guidance for the implementation of green consumption behaviors.	
Green Product-Related Factors	Green Product Packaging	Green product packaging contributes to environmental protection.	De Angelis et al. (2017); Montoro-Rios et al. (2008)
		Green product packaging is an excellent example of eco-friendly packaging.	
		Green product packaging aligns with the environmental positioning of the product.	
		Green product packaging should feature green product labels.	
	Green Brands	The environmental benefits of green brands, such as energy-saving and zero emissions, attract me.	Czellar and Palazzo (2004); Jamal and Al-Marri (2007); Chang and Chen (2008)
		The green manufacturing processes and green technologies of green brands are very appealing to me.	
		Green brands’ products do not harm the environment after disposal, which is very appealing to me.	
		Purchasing products from green brands is beneficial for the environment and society, which is very appealing to me.	
		The values of green brands promoting environmental benefits align with my beliefs.	
		I would feel regret if a brand does not have eco-friendly features.	
		The green corporate culture of the brand’s parent company inclines me towards it.	
		The brands of products I purchase have consistently been green brands.	
		I often recommend green product brands to relatives and friends.	
Personal Factors	Self-Efficacy	I have confidence in implementing green consumption behaviors.	Riggs and Knight (1994); Venkatesh et al. (2003); Lin et al. (2008)
		I believe I have the ability to implement green consumption behaviors.	
		I believe that, as long as I am willing to make an effort, I can improve or address certain environmental issues.	
		If I encounter problems in implementing green consumption behaviors, I have confidence in finding solutions.	
	Ecological Values	Natural resources are the foundation for human survival and development, and humans should reduce their consumption.	Dunlap and Van Liere (1978); Schwartz and Bardi (2001)
		Nature has inherent intrinsic value, and humans should adapt to and respect nature.	
		Human society must coexist harmoniously with nature to survive.	
		Economic development must proceed in parallel with environmental protection.	
		Harmony between humans and nature is a sign of social progress.	
		I believe that caring for nature and protecting the environment is important.	
	Nature Connectedness	I feel connected to the surrounding natural world.	Perrin and Benassi (2009)
		I can perceive and appreciate the wisdom of other life forms.	
		I often feel an affinity with animals and plants.	
		I have a deep understanding of how my actions affect the natural world.	
		I feel that all residents of the Earth (both human and non-human) share a common vitality.	
Emotional Factors	Guilt	If my failure to engage in green consumption leads to adverse environmental impacts, I feel guilty.	Onwezen et al. (2013)
		If my failure to engage in green consumption leads to adverse environmental impacts, I feel remorse.	
		If my failure to engage in green consumption leads to adverse environmental impacts, I feel remorse.	
		If my failure to engage in green consumption leads to adverse environmental impacts, I feel ashamed.	
	Pride	I consider myself an environmentally conscious consumer.	Whitmarsh and O’Neill (2010); Ivanova et al. (2019); Khare and Pandey (2017); Confente et al. (2020)
		The green products I use effectively demonstrate that I am a responsible consumer.	
		I take pride in being an environmentalist.	
		I desire for my family and friends to see me as someone who cares about environmental issues.	
		If I purchase green products, I am very satisfied with myself.	
		I buy and use green products because they highlight my pro-environment personality.	
		Engaging in environmental activities is an important part of my life.	
	Awe	I feel awe towards nature.	Shiota et al. (2006)
		My surroundings are full of beauty.	
		I often seek patterns in the things around me.	
		I have many opportunities to appreciate the beauty of nature.	
		I seek certain experiences to challenge my understanding of the natural world.	
Green Consumption Behavior		I will persuade people around me to upcycle old items or recycle them for environmental purposes.	Ceylan (2019); H. S. Kim and Damhorst (1998)
		If I need to buy shopping bags at the supermarket in the future, I will choose biodegradable shopping bags.	
		When purchasing products, I will select those with minimal environmental pollution.	
		Compared to disposable items, I will choose products that can be reused.	
